# Quality of care of Egyptian asthmatic children: Clinicians adherence to asthma guidelines

**DOI:** 10.1186/1824-7288-36-33

**Published:** 2010-04-21

**Authors:** Ashraf A Salama, Ahmed A Mohammed, El Sayed E El okda, Rasha M Said

**Affiliations:** 1 Department of Pediatrics, Faculty of Medicine, Ain Shams University, Abssia, Cairo, Egypt; 2 Department of Community Medicine, Faculty of Medicine, Ain Shams University, Abassia, Cairo, Egypt

## Abstract

**Background:**

Despite the development and dissemination of guidelines for the diagnosis and management of asthma, a gap remains between current recommendations and actual practice.

**Objectives:**

To assess the physicians attitude towards asthma guidelines and their adherence to its recommendations.

**Methods:**

Three hundred and fifty two clinicians (101 General practitioners, 131 pediatric specialists, 35 pediatric consultants and 85 doctors did not report the qualification) engaged in direct childhood asthma care in Cairo, Egypt were subjected to a self-administered questionnaire with 35 questions of which most were multiple choices, aiming at assessment of three important aspects about the involved physicians; physician's knowledge, practice and attitude. 165 of the clinicians were working in governmental hospitals, 68 clinicians work in private clinics and 119 clinicians work in both.

**Results:**

Agreement with asthma guidelines was present in 76.2% of the studied physicians, however those who not in agreement with the guidelines claimed that this was mainly due to patient factors, firstly the poor socioeconomic standard of the patient (18.1%) and secondly due to poor patient compliance (16%). Poor knowledge was found in 28.5%, poor practice was found in 43.6% and poor attitude was found in 14.4% of the studied physicians. There was positive highly significant correlation between qualification and knowledge, (p < 0.01), positive highly significant correlation between qualification and practice, (p < 0.01), and positive highly significant correlation between qualification and attitude, (p < 0.01).

**Conclusion:**

The attitude of the studied physicians revealed agreement of their majority with the guidelines, while the disagreement was mainly explained by the poor socioeconomic standard of the patients. The degree of poor practice is more marked than that of poor knowledge or poor attitude reflecting resources limitations and applications obstacles in the physician's practice.

## Introduction

Bronchial asthma is a chronic disease of respiratory tract constituting a serious public health problem all over the world [1]. Asthma prevalence has increased very considerably in recent decades such that it is now one of the commonest chronic disorders in the world [2].

Asthma is estimated to affect 300 million people worldwide, with an expected increase to 400 million worldwide by 2025 [3]. In a population of children and adolescents, bronchial asthma occurs with frequency of 5-10% [1]. A chronic inflammatory disease of the airways, asthma causes 0.25 million deaths annually and substantial socioeconomic burden around the globe [3].

A previous study done in Cairo, Egypt to ascertain the prevalence of asthma among children revealed that the overall prevalence of wheezing in the last year was 14.7% and of physician-diagnosed asthma was 9.4%. This study clearly shows that asthma symptoms are much more prevalent among those from poorer backgrounds. Children attending state schools also showed a higher prevalence of severe asthma symptoms but were much less likely to have a physician diagnosis of asthma, which points to discrepancies in access to healthcare. Asthma is relatively common, and probably underdiagnosed and undertreated, particularly among children from less wealthy families [4].

Many factors may have contributed to the rise of the problem of bronchial asthma. Increasing air pollution, fast modernization, and widespread construction work are some of the reasons for asthma to thrive. The situation is complicated by poor access to medical services, high price of effective drugs, and poor health education among the affected population [5].

In spite of these laudable efforts to improve asthma care over the past decade, a majority of patients have not benefited from advances in asthma treatment and many lack even the rudiments of care partly due to the disease entity or patients' compliance, and partly due to physicians' knowledge and disposition in terms of treatment [6].

The number of asthmatic children in any population is far greater than what can be managed by trained persons. Therefore, a vast majority of these children are managed by general or family physicians all over the world. The undergraduate teaching of bronchial asthma is usually confined to one or two lectures and an occasional case-discussion during the clinical case teaching. The general physicians are, therefore, often handicapped in dealing with patients with bronchial asthma. As a result, asthma continues to be underdiagnosed and undertreated [7].

Updated guidelines on bronchial asthma have been recently issued by two major international bodies, namely, GINA (Global Initiative for Asthma) and NAEPP (National Asthma Education and Prevention Program) [8]. Both have not addressed the problems related to developing countries [3,8].

GINA has published evidence-based asthma guidelines since 2002; annual updates are available on the GINA website [9]. These guidelines emphasize the fact that asthma is a chronic inflammatory disorder of the airways and that, while asthma exacerbations are episodic, airway inflammation is chronically present. Exposure to allergens is listed as a common risk factor. The fact that medication must be taken every day by most patients to control symptoms, to improve lung function, and to prevent attacks is noted. Criteria defining the control of asthma are presented in the guidelines. Moreover, the guidelines provide criteria for determining asthma severity; and recommendations for pharmacological management of asthma are outlined according to a stepwise approach based on asthma severity [3,9].

## Aim of the Work

The aim of this study was to assess the current situation as regard clinician attitude towards national and international guidelines for management of pediatric asthma and their adherence to its recommendations through written questionnaire.

## Subjects & Methods

### Type of the study

Cross-sectional study.

### Subjects

Physicians meeting the following criteria were identified:

(1) Engaged in direct childhood asthma care.

(2) Practice location in Egypt.

(3) General practitioners, general pediatrician or pediatrician with subspecialty in pediatric allergy and immunology, pediatric critical care, or pediatric pulmonology.

### Sample size

EPI - Info - program was used for calculation of sample size as follows:

• Power of the test: 80%.

• Confidence interval = 95%.

• Alfa error = 5%.

• Total number of cases selected = 352 physicians.

### Sampling method

Type of sample is simple random sample.

### Tool of the study

A self-administered questionnaire with 35 questions of which most were multiple choices, aiming at assessment of three important aspects about the involved physicians; physician's knowledge, practice and attitude. It covered mainly the following items:

(1) Demographic information about the respondents and their practice settings.

(2) Asthma diagnosis.

(3) Clinical monitoring.

(4) Pharmacological and non-pharmacological treatment of asthma.

(5) Opinions and beliefs about treatment options and reasons for referrals.

(6) Involvement in continuing medical education.

(7) Use of asthma practice guidelines.

(8) Characteristics of practice settings.

(9) Follow up of patients.

(10) Education of asthmatic patients.

### Ethical considerations

1) Confidentiality of information was guaranteed.

2) Anonymous questionnaire.

3) Verbal consent was obtained from each candidate before each questionnaire.

### Statistical methodology

Analysis of data was done by IBM computer using SPSS (statistical program for social science version 12) as follows: Description of quantitative variables as mean, SD and range. Description of qualitative variables as number and percentage. Chi-square test was used to compare qualitative variables between groups. For all tests a probability (p) less than 0.05 was considered significant, less than 0.001 was considered highly significant, more than 0.05 was considered insignificant.

Assessment of the questionnaire on the basis of the licked answers, if more than 50% of the question was answered right the question is considered right and takes a mark, all marks are collected to follow a scoring system using a quartile method as follows: **<**50% = poor. 50% - 75% = moderate. **>**75% = good.

Assessment of knowledge based on answers of questions number 7-19, assessment of practice based on answers of questions number 20-33, assessment of attitude based on answers of questions number 34-35 [10].

## Results

257 of the studied physicians (73%) did hear about asthma guidelines, 186 (52.8%) only did read it. 220 physicians (62.5%) reported that asthma can be prevented by avoidance of introducing solid food to infants below 6 months, 279 physicians (79.3%) reported that it can be prevented by promoting breast feeding, 177 physicians (50.4%) reported that it can be prevented by avoidance of day care nurses, 282 physicians (80.1%) reported that it can be prevented by avoiding maternal smoking, 47 physicians (13.4%) reported that it can be prevented by using long courses of antibiotics, on the other hand 70 physicians (19.9%) reported that asthma is not a preventable disease.

342 physicians (97.2%) reported that diagnosis of asthma basically depends on clinical assessment, 137 (38.9%) reported that diagnosis of asthma basically depends on laboratory investigations, 112 (31.8%) reported that diagnosis of asthma basically depends on radiological investigations, and 253 (71.9%) reported that diagnosis of asthma basically depends on pulmonary function testing.

319 physicians (90.6%) were aware about the use of controllers and relievers in different grades of asthma except mild intermittent, 299 physicians (84.9%) were aware about the correlation between inhaled steroid dose and asthma severity, 239 physicians (67.9%) were aware about the additive effect of leukotriene modifiers and long acting beta II agonists to the anti-inflammatory effect of steroids. However 153 physicians (43.5%) believed that steroid inhalation therapy can significantly affect child's growth.

In practice, 331 physicians (94%)depend on clinical diagnosis of asthma, 156 physicians (45%) claimed that they know how to use PEFM, however only 102 physicians (29.6%) recommended it regularly for diagnosis of asthma and 144 (41%)of the studied physicians stated that they use skin testing for diagnosing asthma (table 1).

**Table 1:** Distribution of the studied group practice as regard asthma diagnosis

	Number	%
On what basis you diagnose a case of asthma in practice?		
• Case with recurrent wheeze and cough	331	94%
• Similar cases in the family is a must	119	33.8%
• Wheezes in the first year of life	126	35.8%
		
Do you know how to use peak flow meter?	156	45.2%
		
Do you recommend it regularly for diagnosis of your patients?	102	29.6%
		
Do you use the following laboratory investigations in diagnosing asthma:		
• CBC	142	40.3%
• Oesinophilic count	244	69.3%
• IgE	177	50.3%
• Skin testing	144	41%

There was significant defect in the studied physicians practice as regard assessment of bronchial asthma severity as 249 physicians (71.1%) were not able to define the degree of severity, however 103 (28.9%) could reasonably assess asthma severity as referring to GINA guidelines.

In their practice, 302 (85.8%) of the studied physicians were using steroids as a controller, however 143 (40.7%) still giving it in the oral form, and 234 (66.5%) were using it as short term therapy as long term therapy in the practice of 140 (39.9%) of the studied physicians and 97 (27.6%) were using the oral form. 245 (72.2%) of the physicians were using cough medications in their asthma management and 187 (53.1%) of them were prescribing antibiotics.

205 (58.2%) of the physicians were using written instructions for their patients and 208 (59.4%) were having a way for communication with their asthma patients; however PEFM was used only by 79 (22.5%) of the physicians for follow up (table 2).

**Table 2:** Distribution of the studied group as regard home management in the practice of the studied physicians

	Number		%	
	30 (+45) days*			
How frequent the follow up for your patient?				
		
Do you use written instructions about symptoms and treatment for your asthma patient?				
Yes	205		58.2%	
No	147		41.8%	
		
Is there a way for communication between you and your patient?				
Yes	208		59.4%	
No	142		40.6%	
		
Are you using PEFM for follow up of your asthma patient?				
Yes	79		22.5%	
No	272		77.55%	

Agreement with asthma guidelines was present in 266 (76.2%) of the studied physicians, however those who not in agreement with the guidelines claimed that this was mainly due to patient factors, firstly the poor socioeconomic standard of the patient 63 (18.1%) and secondly due to poor patient compliance 56 (16%). Patient education was important in their opinion whether recognizing symptoms 318 (91.1%), allergen triggers 333 (95.4%) or the use of medications, but it comes down to 176 (50.3%) in using PEFM.

Poor knowledge was found in 28.5%, poor practice was found in 43.6% and poor attitude was found in 14.4% of the studied physicians (table 3).

**Table 3:** Distribution of the studied physician's knowledge, practice and attitude

	Number	%
Poor knowledge	100	28.5
		
Poor practice	153	43.6
		
Poor attitude	50	14.4

Correlation between qualification and different aspects evaluating physician's adherence to asthma guidelines revealed that: there was positive highly significant correlation between qualification and knowledge; p < 0.01 (table 4), positive highly significant correlation between qualification and practice; p < 0.01 (table 5), and positive highly significant correlation between qualification and attitude; p < 0.01 (table 6).

**Table 4:** The relation between qualification and knowledge

	GP	Specialist	Consultant	X 2	P
Poor knowledge	9 (8.9%)	10 (7.6%)	1 (2.9%)		
					
Moderate knowledge	63 (62.4%)	46 (35.1%)	9 (25.7%)	28	<0.01 HS*
					
Good knowledge	29 (28.7%)	75 (57.3%)	25 (71.4%)		

*Highly Significant

**Table 5:** The relation between qualification and practice

	GP	Specialist	Consultant	X^2^	P
Poor Practice	58 (57.4%)	60 (45.8%)	11 (31.4%)		
					
Moderate practice	36 (35.6%)	38 (29%)	9 (25.7%)	24	<0.01 HS*
					
Good practice	7 (6.9%)	33 (25.2%)	15 (42.9%)		

*Highly Significant

**Table 6:** The relation between qualification and attitude

	GP	Specialist	Consultant	X^2^	P
Poor attitude	51 (50.5%)	20 (15.3%)	1 (2.9%)		
				47.8	<0.01 HS*
Good attitude	50 (49.5%)	111 (84.7%)	34 (97.1%)		

*Highly Significant

## Discussion

This study was carried out to assess the current situation as regard clinician awareness and attitude towards national and international guidelines and their adherence to its recommendations.

In this study a written questionnaire with different topics were added to achieve our study objective, this questionnaire was fulfilled by 352 clinicians, all of them deal with childhood asthmatics, the mean age of patients they manage was 7-10 years old, 101 were general practitioners, 131 pediatric specialist and 35 pediatric consultants and 85 doctors did not report the qualification, 165 of the clinicians were working in governmental hospitals, 68 clinicians work in private clinics and 119 clinicians work in both.

Knowledge assessment in the studied physicians group revealed that the most important source for information about the guidelines was lecture attendance (73%), active reading of the guidelines observed in 52.8% only of the physicians, this reflects the importance of continuous medical education programs in supplying the physicians with up to date knowledge. On the other hand it also reflects a limited capacity of the physician for self education.

In spite of awareness of the importance of breast feeding (79.3%) in asthma prevention, and the role of maternal smoking in its aggravation, 13.4% of the studied physicians still believe that long courses of antibiotics can prevent asthma, this points to the fact that adding new concepts is much more easier than changing the old ones [11].

In agreement a survey by *Gharagozlou et al. (2008) *in Iran reported that awareness about the standard guidelines among the Iranian pediatricians who participated in the survey was low [12]. However different studies showed that continuing medical education can increase the knowledge of physicians after graduation especially in younger physicians [13].

The awareness of using PEFM in diagnosing asthma was high (71.9%), however, when coming to the practice of the physicians only 29.6% of them use it in their practice for diagnosing asthma, moreover, only 22.5% of the physicians use PEFM for follow up of the patients.

This was in agreement with *Gharagozlou et al. (2008) *where they found only a minority of pediatricians use PEFM as a part of regular care of their patients [12], *Warman et al. (1999) *also found that only 30% of the children with persistent asthma had PEFMs [14].

Although most of the physicians agreed that corticosteroids should be used as controller (85.8%) and the dose should be tailored according to asthma severity (84.9%), still 43.5% of them believe that this therapy affects the patient's growth significantly. This belief affects practice of the physician in prescribing inhalation corticosteroids as a controller only for short term therapy (66.5%), this lack of good knowledge may create an important obstacle in the physician's adherence to the recent guidelines which mandated controller therapy for long periods according to the degree of control.

This was on the contrary with the study done in Iran which revealed that only one third of the Iranian pediatricians prescribed inhaled anti-inflammatory (corticosteroids) for these cases. The reasons as they reported for this mismanagement were existence of better choices for treatment of asthma and also the fear of the long term side effects of inhaled corticosteroids [12]. The prescription of such inhalers for similar patients was more than 80% by the studied physicians in Turkey [15].

Fixed beliefs about food elimination in patient with bronchial asthma without clear evidence for its role in pathogenesis of asthma might predispose to an important defect in our studied physician's practice.

*Prochaska and DiClemente (1983) *suggested that most of the physicians surveyed in their study were in a precontemplation stage where they were not ready yet to change their behavior [16].

Severity assessment in the practice of the studied physicians was significantly lacking, 71.1% of them do not know how to assess asthma severity. This might be a great obstacle in managing asthma cases and in following the guidelines, not only because of the lack of proper assessment but also because inappropriate controller dose selection according to the degree of severity.

Physicians may not be able to overcome the inertia of previous practice or they may not have the motivation to change [16]. The practice of prescribing bronchodilators as long term therapy still present in 39.9% of the physicians and 27.6% of them prescribe it in syrup form, moreover, 53.1% of the physicians were prescribing antibiotics routinely as a therapy for childhood asthma, which is in disagreement with the recommendations of the guidelines.

Written instructions for the patients were given by 58.2% of physicians to the patients. However the questionnaire was not stressing on whether it was in an action plan form or not.

Agreement with the guidelines was relatively adequate in the studied physicians (76.2%), disagreement was mainly due to the poor socioeconomic standard of the patients as claimed by the physicians.

Similarly *Christakis and Rivara (1998) *surveyed the pediatrician's attitude towards guidelines and they reported barriers to adherence mainly lack of agreement with specific recommendations [17].

Patient education is also an essential component of asthma care. The NAEPP guidelines suggest that in addition to education delivered by the clinician, all patients may benefit from formal asthma education programs. This study showed that most of our participants agree with the concept of patient education, patient education should include how to use medication (97%), how to recognize allergen triggers (95%), how to recognize and report asthma symptoms (91%) and lastly how to use PEFM (50%).

Poor practice (43.6%) in the studied physicians was more marked than poor knowledge (28.5) or poor attitude (14.4%). *Cloutier et al. (2002) *stressed on the impact of education interventions on physicians caring for patients with asthma in poor urban areas which is suboptimal because of limited resources and barriers to deliver good quality of care [18].

In general when it comes to compare physician's knowledge, practice and attitude most of the physicians had good knowledge and attitude in comparison to their practice, although there was a significant correlation between all of them, in our study we revealed that knowledge, practice and attitude have no relation with practice location whether it is governmental or private place, but they were greatly related to qualification.

Our results were consistent with previous studies that have shown inconsistency of care with guidelines for children with asthma and advantages of specialists in the delivery of asthma care, especially in patients with previous acute health care encounters [19].

*Doerschug et al. (1999) *demonstrated that asthma specialists performed better than general and family physicians on a multiple-choice test about asthma guidelines in areas of pharmacology and prevention [20].

A cross-sectional study in a large managed care organization showed that most patients with moderate or severe asthma symptoms treated by allergists received anti-inflammatory therapy (92%), compared with less than half of patients of generalists (42%) [21].

In a study of patients who had used an emergency department and were then randomly assigned to allergist referral versus usual care, the patients in the allergist group were 3 times more likely to use anti-inflammatory medications and had a 42% reduction in subsequent emergency department use over 6 months [22].

In another study of children cared for by allergists versus primary care physicians, the patients of allergists had better quality of life. Reasons for these differences in outcome were not examined. Better asthma care may result from better knowledge of treatment elements included in national asthma guidelines [23].

*Harrold et al. (1999) *reported that specialists have more knowledge of selected conditions, were more likely to use medications associated with better survival, were more likely to comply with screening guidelines, and achieved better outcomes for some but not all disorders [24].

*Gregory et al. (2001) *also noted that specialists use more resources, including ordering more tests and having greater inpatient length of stay [25]. Generalists, on the other hand, perform better on tests of general medical knowledge and do better in some areas of health promotion and disease prevention, risk behavior counseling, and recognition and management of psychosocial problems [26].

## Limitations

There are several limitations to this study. Our survey response rate was only about 55-60% and respondents were more likely to be general practitioners or master-certified in pediatrics. Both factors may affect the generalizability of our results to practicing pediatricians.

This study's generalizability is also limited by how participants were selected. Volunteers were recruited several CME conferences and may represent practices that are more affluent or conscious of staying up-to-date. The sessions were also conducted at an academic setting, which may influence participants to give "correct" or "professionally acceptable" responses, like other self-reported data, respondents may have reported what they believed to be acceptable, instead of their actual practice

Adherence was based on self-report, which might not reflect actual adherence. Studies of physician practice suggest that physician self-report can overestimate or underestimate actual practice when compared with chart audits or patient surveys [27].

The analysis assumes that self-report bias will affect measures of barriers to adherence as well as guideline adherence, thus preserving the profile of barriers for each guideline component. It is also encouraging that few respondents reported familiarity with the spurious guideline components that we included to test the validity of self-report.

Results from other studies suggest that physician self-report is not wildly misleading. For example, *Diette et al (2001) *surveyed 318 parents of children with asthma and found that 55% of patients with daily asthma symptoms used a daily controller medication compared with 36.5% in our study [28]. *Warman et al (1999*) surveyed 220 parents of children with asthma and found that only 30% of children with persistent asthma had PEFMs [14]. In our sample, self-reported adherence rate for instruction of PEFM use was similar at 29.6%.

And lastly these findings reflected asthma care at a single point of time. This study highlighted a lot of differences between the recommended management of asthma and the primary care which is carried out in our society. An understanding of the reasons for these differences can help us to decrease the number of mismanaged asthmatic patients.

***In conclusion***, the attitude of the studied physicians revealed agreement of their majority with the guidelines, while the disagreement was mainly explained by the poor socioeconomic standard of the patients.

The lack of the physicians adherence to the guidelines was due to lack of awareness and familiarity about some specific aspects of the guidelines mainly the use of PEFM in diagnosis, management and follow up, the use of corticosteroid inhalation therapy as a long term controller therapy and the use of inhalation bronchodilator therapy on demand.

Also inertia of the previous practice in prescribing antibiotic courses, oral bronchodilators and food elimination may also prevent adherence of the studied physicians to guidelines, lack of self efficacy and lack of agreement may also be minor determinant in this non-adherence.

Knowledge of the physicians was found to be derived mainly from attending lectures rather than active readings, knowledge, practice and attitude are strongly related to the level of qualification which seems to be a good motivation for acquiring recent knowledge and improving the physician's practice, however, the degree of poor practice was more marked than that of poor knowledge or poor attitude reflecting resources limitations and applications obstacles in the physician's practice.

### Finally

Asthma represents an increasing health burden for Egypt. Health care delivery is inadequate in Egypt. Irrational prescribing and practices are generally widespread and misconceptions are commonly encountered, both among the public and health professionals. Minimum standards of health care for individuals with asthma need to be identified.

### Recommendation

• Public education about asthma is urgently required.

• Training of health care professionals on the management and control of asthma is very important.

• Opportunities for primary prevention should be promoted and facilitated.

• Preparation of a "model" standardized protocol for an epidemiological study that is relevant to the region is mandatory.

• Formulate guidelines for national plans for asthma control which also cover aspects related to implementation and evaluation.

• Adapt the international guidelines for asthma management to suit regional needs and circumstances.

## Competing interests

The authors declare that they have no competing interests.

## Authors' contributions

AAS, AAM and RMS conceived the study design, EEE performed the statistical data analysis, and AAM wrote the manuscript. All authors have read and approved the final manuscript.

## Appendix

QUESTIONNAIRE

NAME (Optional):

AGE (Optional):

(1) QUALIFICATIONS:

(2) SPECIALITY:

SUBSPECIALITY:

(3) PRACTICE LOCATION:

• Governmental Yes { } No { }

• Private Yes { } No { }

(4) *Do you manage patients with bronchial asthma in your practice? Yes { } No { }*

If yes:

(5) *What is the age range of the patients ( )*

(6) *Do you consider yourself able to manage childhood asthma?*

• Perfectly { }

• Adequately { }

• Inadequately { }

(7) *Do you usually attend scientific meetings, CME programs concerning childhood asthma Yes { } No { }*

If yes; how frequent

• Monthly Yes { } No { }

• Annually Yes { } No { }

• More than one year intervals Yes { } No { }

(8) *Did you hear about asthma guidelines? Yes { } No { }*

If yes:

*Did you read it before? Yes { } No { }*

If yes:

*When were the most recent guidelines? ( )*

*What is the source of your information?*

• Lecture Yes { } No { }

• Printed form Yes { } No { }

• On line Yes { } No { }

(9) *In your opinion; is asthma a preventable disease? Yes { } No { }*

If yes:

*Asthma can be prevented by:*

• Avoidance of introduction of solid food to infants up to age of 6 months Yes { } No { }

• Promotion of breast feeding Yes { } No { }

• Avoidance of admitting the child to day care nurses Yes { } No { }

• Avoidance of maternal smoking Yes { } No { }

• Using long courses of antibiotics Yes { } No { }

(10) *What is your opinion about asthma?*

• It is due to inflammatory process in the airways Yes { } No { }

• It is just recurrent and reversible bronchoconstriction Yes { } No { }

• It is prevalent in all age groups including infancy Yes { } No { }

• Genetic factor have important role in asthma Yes { } No { }

(11) *Diagnosis of bronchial asthma basically depends upon:*

• Clinical assessment Yes { } No { }

• Lab. Investigations Yes { } No { }

• Radiological investigations Yes { } No { }

• Pulmonary functions test Yes { } No { }

(12) *Grading of bronchial asthma severity is based upon:*

• Frequency of diurnal symptoms Yes { } No { }

• Frequency of nocturnal symptoms Yes { } No { }

• Peak expiratory flow meter measurements Yes { } No { }

(13) *The aim of asthma control is:*

• The child can lead a normal life without activity limitation Yes { } No { }

• To have no symptoms at all Yes { } No { }

• To reduce acute exacerbations and need for hospitalization Yes { } No { }

• To normalize lung functions Yes { } No { }

(14) *Treatment of bronchial asthma should include:*

• Relievers (e.g. ventoline) and controllers (steroids) except in mild intermittent asthma Yes { } No { }

• Controllers should be used for long term therapy with empiric dose unrelated to asthma severity Yes { } No { }

• Dose of steroids should be defined to asthma severity Yes { } No { }

• Addition of other drugs like montelukast or long acting beta 2 agonist can reduce steroid inhalation dose Yes { } No { }

• Steroid inhalation therapy can affect significantly child's growth Yes { } No { }

(15) *The use of relievers inhalation therapy (ventoline) is essential to:*

• Control disease activity Yes { } No { }

• Control symptoms Yes { } No { }

• Should be maintained for long time Yes { } No { v }

(16) *Immunotherapy:*

• Is always effective in asthma control Yes { } No { }

• Should be done for selected patients Yes { } No { }

• Is very dangerous in children Yes { } No { }

(17) *Environmental control should include:*

• Avoidance of allergen triggers (eg. Dust mite, pollens & molt) Yes{ } No{ }

• Elimination of list of food (eg. Egg, fish, chocolate & strawberry)should be done in each patient Yes { } No { }

(18) *Recognition of the allergen triggers of asthma can be done by:*

• Skin prick test Yes { } No { }

• Specific IgE Yes { } No { }

• In vitro RAST Yes { } No { }

(19) *On what basis you diagnose a case of bronchial asthma in your practice?*

• Case with recurrent wheeze & cough Yes { } No { }

• Similar cases in the family is a must Yes { } No { }

• Wheezes in the first year of life Yes { } No { }

(20) *Do you know how to use peak flow meter?*

If yes;

*Do you recommend it regularly for diagnosis & follow up of your patients? Yes { } No { }*

(21) *Do you use the following laboratory investigations in diagnosing asthma?*

• CBC Yes { } No { }

• Oesinophilic count Yes { } No { }

• IgE Yes { } No { }

• Skin testing Yes { } No { }

(22) *How can you assess the degree of severity of your asthma patient?*

• I don't know ( )

*A) When the frequency of diurnal symptoms (cough, expectoration, wheezes) is:*

• ( )/month consider mild intermittent

• ( )/week consider mild persistent

• ( )/week consider moderate persistent

• ( )/week consider severe persistent

*B) When the frequency of the nocturnal symptoms is:*

• ( )/month consider mild intermittent

• ( )/week consider mild persistent

• ( )/week consider moderate persistent

• ( )/week consider severe persistent

*C) Peak expiratory flow meter reading is:*

• ( )% mild intermittent

• ( )% mild persistent

• ( )% moderate persistent

• ( )% severe persistent

(23) *You can consider the patient controlled when:*

• The frequency of symptoms less than ( )/week

• Using the relievers less than ( )/week.

(24) *In your practice; you prescribe medications according to:*

• Degree of asthma severity Yes { } No { }

• Degree of asthma control Yes { } No { }

(25) *Do you prescribe steroids as controller medications: Yes { } No { }*

If yes:

Do you use them as?

• Short term therapy to control symptoms Yes { } No { }

• Long term therapy to control symptoms of disease activity Yes { } No { }

(26) *What form do you prefer?:*

• Oral Yes { } No { }

• Inhalation Yes { } No { }

(27) *Do you use beta 2 sympathomimetics (ventolin) as:*

• Long term therapy Yes { } No { }

• Short term therapy Yes { } No { }

• On demands Yes { } No { }

(28) *Do you prefer to use them as:*

• Oral form Yes { } No { }

• Inhalation form Yes { } No { }

(29) *Do you use other medications in treating asthma like:*

• Ketotifen, (zaditine) Yes { } No { }

• Antihihistaminics Yes { } No { }

• Cough medications Yes { } No { }

• Antibiotics Yes { } No { }

• Leukotriene antagonists (Montelucast) Yes { } No { }

• Long acting beta II agonists (servent) Yes { } No { }

(30) *Do you use written instructions about symptoms and treatment for your asthma patient? Yes { } No { }*

(31) *How frequent the follow up for your patient? ( )*

(32) *Is there a way for communication between you and your patient? Yes { } No { }*

(33) *Are you using peak flow meter for follow up of your asthma patient? Yes { } No { }*

(34) *Do you agree with asthma guidelines? Yes { } No { }*

If no, why do you disagree?

• Because it's not applicable in practice Yes { } No { }

• Because of the poor socioeconomic standard of the patient Yes { } No { }

• Because it's not workable (results are not good) Yes { } No { }

• Because the treatment should be tailored according to the status of each patient Yes{ } No{ }

• Because of the poor patient compliance to the guidelines Yes { } No { }

(35) *Patient education should include:*

• How to recognize & report asthma symptoms Yes { } No { }

• How to recognize allergen triggers and avoid themYes { } No { }

• How to use the medications Yes { } No { }

• How to use the peak flow meter Yes { } No { }
